# An improved and efficient mutual authentication scheme for session initiation protocol

**DOI:** 10.1371/journal.pone.0213688

**Published:** 2019-03-28

**Authors:** Yuanyuan Zhang, Kunming Xie, Ou Ruan

**Affiliations:** School of Computer Science, Hubei University of Technology, Wuhan, CHINA; King Saud University, SAUDI ARABIA

## Abstract

Qiu et al. made a security analysis about the protocols of Chaudhry et al. and Kumari et al. in 2018, and they pointed out that there are many security weaknesses in the protocols. To improve the security, Qiu et al. proposed an advanced authentication scheme for Session Initiation Protocol on the basis of the previous protocols and claimed that their own protocol was very secure and practical. However, we demonstrate that the protocol of Qiu et al. has a serious mistake which causes their protocol cannot be executed normally. Beyond that, we also find out that their protocol cannot withstand insider attack and denial service attack. In order to remove these weaknesses, we propose an efficient provably secure mutual authentication scheme. Furthermore, our scheme provides security analysis with the help of Burrows-Abadi-Needham (BAN) logic. Compared with their protocol, ours has greater security and better performance.

## 1 | Introduction

With the network of universal gradually, we often send messages, make phone calls and watch videos via the network, which brings great convenience to our lives. However, the network security issues become increasingly prominent. In order to enhance the security of the Session Initiation Protocol (SIP) in communication via the network, lots of scholars have proposed numerous solutions. The first authentication scheme about SIP on the basis of Hypertext Transport Protocol (HTP) authentication was proposed in 1999 [1], but Yang et al. [2] proved that the scheme was insecure in 2005, then they proposed an improved scheme. In fact, the scheme of Yang et al. still has security loopholes. Latterly, although some scholars want to design secure and practical schemes [3–11], the most schemes have more or less flaws.

In recent years, Zhang et al. [12] proposed a remote mutual authentication protocol with protecting user anonymity for SIP, but there are also lots of loopholes in their protocol too. It was as expected that Lu et al. [13] showed that their protocol lacks mutual authentication and cannot resist insider attack in 2016. In order to remove these weaknesses, Lu et al. proposed a new scheme protocol based on the protocol of Zhang et al., but it still had fatal weaknesses in security. Chaudhry et al. [14] found that the scheme of Lu et al. is invalid to impersonation attack. Besides, Kumari et al. [15] showed that the scheme of Lu et al. cannot withstand identity guessing attack and forgery attack. According to their conclusion, Chaudhry et al. proposed a mutual authentication protocol, Kumari et al. proposed an authentication protocol too. However, on the basis of the schemes of Chaudhry et al. and Kumari et al., Qiu et al. [16] demonstrated their schemes are frail for some attacks including insider attack, off-line guessing attack and so on. To overcome these defects, Qiu et al. proposed an improved mutual authentication scheme, claiming that the scheme not only could resist above certain attacks, but also adopted a method of generating random numbers instead of time labels to solve the problem that time is difficult to synchronize. We have to admit that the improved scheme of Qiu et al. did remove certain weaknesses. But after our analysis, we find that the scheme of Qiu et al. has a serious mistake which make the scheme cannot execute properly and cannot resist some attacks. Considering the worst condition, the adversary affects user password update even accesses crucial information of a legal user. In order to overcome the weaknesses, we design a new, more secure and high-performance scheme.

We will focus on the rest seven sections to describe our paper, section 2 reviews Qiu et al.’s scheme. In section 3 we pay attention to analyze the weaknesses about Qiu et al.’s scheme. Section 4 describes our proposed scheme. Next section 5 and section 6 mainly provide analysis and proof of security. Section 7 gives the performance comparison among our scheme and the relative schemes of other scholars. Finally, section 8 shows the conclusion of our scheme.

## 2 | Review of the scheme of Qiu et al.

In this section, we will briefly review Qiu et al.’ s scheme [16], which contains three phases: registration phase, login and authentication phase and password update phase. The detailed information about the three phases is shown as follows.

Before we show each phase, the notations which throughout this paper are introduced in Table 1 firstly.

**Table 1:** The notations of the schemes.

Symbol	Description
***U***	A legitimate user
***S***	The remote server
***Id***	The identity of the *U*
***Pw***	The password of the *U*
***Up***	The secret key of the *U*
***S***_***p***_	The private key of the *S*
***Q***_***s***_ ***= S***_***p***_*·****P***	The public key of the *S*
***h*(·)**	A one-way hash function
**||**	String concatenation operation
**⊕**	The bitwise XOR operation
***sk***	The session key between *U* and *S*

### 2.1| Registration phase

During the registration phase, user *U* and server *S* do the following operations to finish registering.

- *U*⇒*S*:{*Id*,*HId*}:
  A user *U* registers to server *S* with his/her identity *Id*, password *Pw* and secret key *Up*. After that, *U* completes *VPw* = *h*(*Pw*‖*Up*), *HId* = *h*(*Id*‖*VPw*) and transmits {*Id*, *HId*} to sever *S*.
- After receiving *Id* and *HId*, *S* will compute *N* =*HId*⊕*h*(*S*_*p*_) and store *N* into database.

### 2.2| Login and authentication phase

If a user *U* completed the registration phase successfully and wants to access request to the server *S*, *U* and *S* should perform the following steps.

- *U*→*S*:{*B*,*X*,*T*}:
  The user *U* accesses server *S* with his or her identity *Id*, password *Pw* and secret key *Up*. After that *U* calculates *VPw* = *h*(*Pw*‖*Up*), generates a long random number *r* and computes *HId* = *h*(*Id*‖*VPw*), *W* = *h*(*Id*‖*Up*), *X* = *r*⋅*P*, *Y* = *r*⋅*Q*_*s*_, *B* = *W*⊕*h*(*HId*‖*Y*) and *T* = *h*(*B*‖*Y*‖*W*). After completion of calculation, *U* sends {*B*,*X*,*T*} to server *S*.
- *S*→*U*:{*E*,*Auth*_*s*_}:
  When server *S* receives message {*B*,*X*,*T*}, it will calculate *HId* = *N*⊕*h*(*S*_*p*_), *Y* = *S*_*p*_⋅*X*, *W* = *B*⊕*h*(*HId*‖*Y*) and check whether T* = *h*(*B*‖*Y*‖*W*) is equal to *T*. If not valid, the session will be terminated by *S*, otherwise server *S* generates a long random number *r*′ and calculates *E* = *r*′⋅*Q*_*s*_, *sk*_*s*_ = *r*′⋅*Y*, *Auth*_*s*_ = *h*(*sk*_*s*_‖*W*‖*Y*). After that, *S* transmits information {*E*,*Auth*_*s*_} to *U*.
- *U*→*S*:{*Auth*_*u*_}:
  Upon getting {*E*,*Auth*_*s*_}, *U* calculates *sk*_*u*_ = *r*⋅*E*,*Auth*_*s*_′= *h*(*sk*_*u*_‖*W*‖*Y*). Then *U* checks whether *Auth*_*s*_′ = *Auth*_*s*_. If valid, then *U* computes *Auth*_*u*_ = *h*(*sk*_*u*_‖*W*‖*Y*‖*E*) and sends result *Auth*_*u*_ to *S*, else terminates the session.
- Once receiving the message {*Auth*_*u*_}, *S* computes *Auth*_*u*_′= *h*(*sk*_*s*_‖*W*‖*Y*‖*E*). If *Auth*′_*u*_ is not equal to *Auth*_*u*_, *S* terminates the session. After that, the user *U* communicates with server *S* based on the common session key *sk* = *sk*_*u*_ = *sk*_*s*_ = *r*⋅*r*′⋅*Q*_*s*_.

### 2.3| Password update phase

For a legitimate user *U*, if he or she wants to change own password *Pw* for some reasons, the following steps will be performed.

- *U*→*S*:{*V*,*M*}:
  *U* already picks a new password *Pw*^*new*^ and a new secret key *Up*^*new*^, after that he or she inputs identity *Id*, password *Pw* and secret key *Up*. *U* computes *V* = *h*(*sk*‖*h*(*Id*‖*h*(*Pw*‖*Up*))) and *M* = *h*(*Id*‖*sk*)⊕*h*(*Id*‖*h*(*Pw*^*new*^‖*Up*^*new*^)), then *U* transmits message {*V*,*M*} to *S*.
- After obtaining message {*V*,*M*}, *S* computes V* = *h*(*sk*‖*N*⊕*h*(*S*_*p*_)) firstly, then *S* judges if the value of V* is equal to *V*. If holds, *S* will replace *N* with *N*^*new*^ by computing *N*^*new*^ = *h*(*S*_*p*_)⊕*h*(*Id*‖*sk*)⊕*M* into database. If not, *S* fails to change password and exits the session.

## 3 | Weakness of scheme proposed by Qiu et al.

In this section, we analyze the weaknesses of Qiu et al.’ s scheme [16] carefully. After our study, we find that their scheme has a serious mistake which causes the scheme cannot executed normally. What is more, their scheme cannot resist insider attack, denial service attack and makes user *U* have poor experience [17].

### 3.1 | Serious mistake

In registration phase of Qiu et al.’s scheme, we notice that information *N* is stored into database alone. As is known to all, there should be some information like identity *Id* correspond to *N* in the database. Or else in login and authentication phase in their scheme, when *S* receives message from users, *S* cannot match corresponding *N* in database without the help of the corresponding information. So, the scheme of Qiu et al. is unable to carry out normally.

Perhaps Qiu et al. just forgot corresponding information, here we help them supply corresponding information on the basis of the scheme of Qiu et al. In registration, *S* only knows information *Id*, *HId* and *N* which relates to *U*. *HId* is the most important secret data during the entire protocol execution process, so the server cannot store *HId* in the database but stores *Id*. We assume a semi honest server *S*_0_ has the ability of gaining and calculating the sensitive information in the sever. If *Id* corresponds to *N*, we notice that an adversary *S*_0_ can obtain {Id*, Up*, Pw*} of a legitimate user U* in login and authentication phase by off-line guessing attack, the specific steps are as follows.

**Step 1:** According to the login and authentication process in the scheme of Qiu et al., *S*_0_ will get the values of HId* and W* at time of calculating HId* = N*⊕*h(S*_*p*_*)* and W* = B*⊕*h*(HId*⊕Y*). Besides, because Id* corresponds to N*, *S*_0_ can get the corresponding value of Id*.

**Step 2:** After getting user *U'*s sensitive information {Id*, HId*, W*}, *S*_0_ can guess the value of Up* from the identity space by calculating W* = h(Id*||Up*). According to the same truth, *S*_0_ can guess the value of Pw* from the identity space by calculating HId* = h(Id*||VPw*) = h(Id*||h(Pw*||Up*)). Obviously, *S*_0_ can access {Id*, Up*, Pw*}.

Through the above steps, *S*_0_ has successfully accessed information {Id*, Up*, Pw*} of a legal user U*.

### 3.2 | Insider attack

In this part, we assume that a malicious insider adversary *A* can obtain some sensitive information in the database of server *S*. In Qiu et al.’s scheme, the adversary *A* can achieve insider attack by registering as a legitimate user. Firstly, he masquerades as a legitimate user in registration to input identity Id*, password Pw* and secret key Up*, then *S* will store the corresponding value N* into database and he can get the value N* form the database of sever *S*. After that *A* has already mastered the information {Id*,Up*,Pw*,N*}. On the basis of formulas *HId* = *h*(*Id*‖*h*(*Pw*‖*Up*)) and *HId* = *N*⊕*h*(*S*_*p*_), *A* can get *h*(*S*_*p*_) = N*⊕*h*(Id*‖*h*(Pw*‖Up*)). In addition, *A* can get other user’s *N*_0_ in the database, so he will get the user’s corresponding *HId*_0_ by formula *HId*_0_ = *N*_0_⊕*h*(*S*_*p*_). In login and authentication phase, *A* can impersonate to be the user *U*_0_ to access sever *S* by corresponding *N*_0_ and *HId*_0_. The specific steps are as follows.

- *U*_0_→*S*:{*B*_0_,*X*_0_,*T*_0_}:
  Adversary *A* chooses random numbers *r*_*0*_, *W*_*0*_ and computes *X*_0_ = *r*_0_⋅*P*, *Y*_0_ = *r*_0_⋅*Q*_*s*_ *B*_0_ = *W*_0_⊕*h*(*HId*_0_‖*Y*_0_) and *T*_0_ = *h*(*B*_0_‖*Y*_0_‖*W*_0_). After completion of calculation, *U*_0_ sends information {*B*_*0*_, *X*_*0*_, *T*_*0*_} to server *S*.
- *S*→*U*_0_: {*E*,*Auth*_*s*_}:
  Once getting message {*B*_*0*_, *X*_*0*_, *T*_*0*_}, *S* computes *HId*_0_′ = *N*⊕*h*(*S*_*p*_), *Y*_0_′ = *S*_*p*_⋅*X*_0_ and *W*_0_′ = *B*_0_⊕*h*(*HId*_0_′‖*Y*_0_′). Obviously, the verification equation *T*_0_′ = *h*(*B*_0_‖*Y*_0_′‖*W*_0_′) is true. Next *S* generates random number *r*′ and computes *E* = *r*′⋅*Q*_*s*_, *sk*_*s*_ = *r*′⋅*Y*_0_′, *Auth*_*s*_ = *h*(*sk*_*s*_‖*W*_0_′‖*Y*_0_′). After that, *S* transmits {*E*,*Auth*_*s*_} to *U*_0_.
- *U*_0_→*S*:{*Auth*_*u*_}:
  When getting {*E*,*Auth*_*s*_}, *U*_0_ computes *sk*_*u*_ = *r*_0_⋅*E*, *Auth*_*u*_ = *h*(*sk*_*u*_‖*W*_0_‖*Y*_0_‖*E*) and sends {*Auth*_*u*_} to *S*.
- After receiving the message {*Auth*_*u*_}, *S* computes *Authu*′ = *h*(*sk*_*s*_‖*W*_0_′‖*Y*_0_′‖*E*) and gets a conclusion that *Auth*′_*u*_ is equal to *Auth*_*u*_. Finally, the user *U*_0_ communicates with server *S* based on the common session key *sk* = *sk*_*u*_ = *sk*_*s*_ = *r*_0_⋅*r*′⋅*Q*_*s*_

We draw a conclusion that *A* can masquerade as an arbitrary legitimate user for entering server *S* by insider attack.

### 3.3| Denial service attack

We assume that an adversary *A* is able to intercept message which is transmitted between *U* and *S*. At password update phase in Qiu et al.’s scheme, *U* sends the result values of *V* and *M* to *S*. The adversary *A* intercepts message {*V*, *M*} and forges *M*_*0*_ by generating random number, then *A* transmits {*V*, *M*_*0*_} to *S*. Apparently, *A* will pass verification of *S* by checking whether *V* is equal to V*. After that, *S* computes *N*^*new*^ = *h*(*S*_*p*_)⊕*h*(*Id*‖*sk*)⊕*M*_0_ and replaces *N* with *N*^*new*^ in the database. Because of the falsify of *N*^*new*^, the user *U* will fail to pass verification in next login and authentication phase.

### 3.4 | Defects of practicality

In term of Qiu et al.’s scheme, we find that a user *U* needs to remember identity *Id*, password *Pw* and secret key *Up*. It is a burden for users to keep in mind with three different data. If a user *U* wants to change password, according to the password update phase of Qiu et al., he or she has to bear in mind with a new password *Pw*^*new*^ and a new secret key *Up*^*new*^. For the most users, it is hard to remember so much information. Therefore, the scheme of Qiu et al. has poor practicability.

## 4 | Our proposed scheme

**Fig 1:**
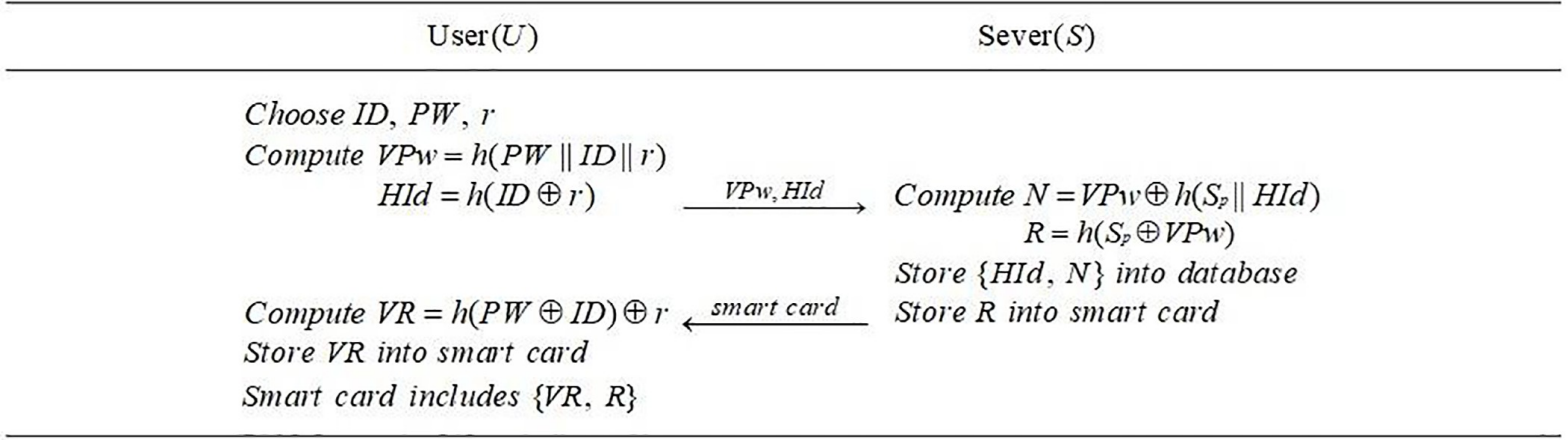
Registration phase.

**Fig 2:**
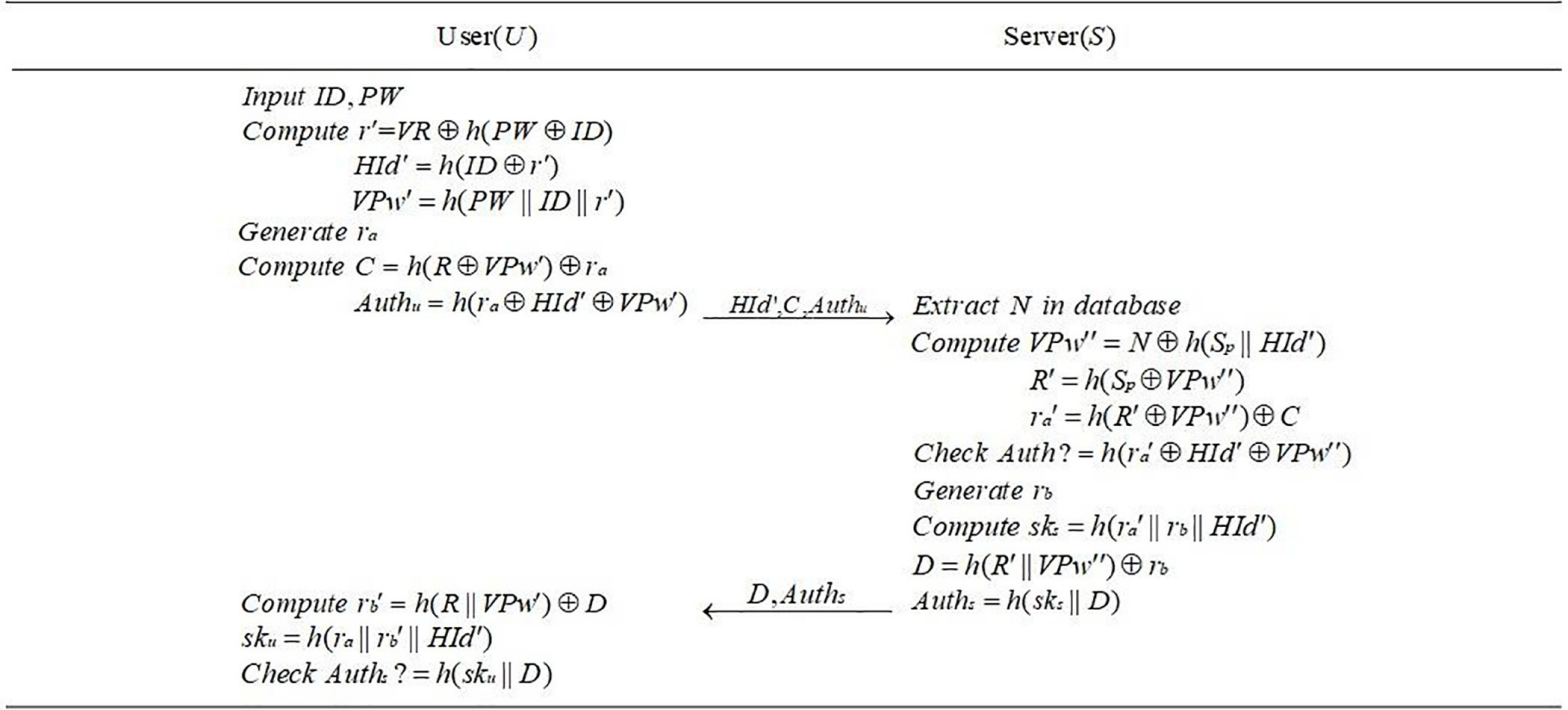
Login and authentication phase.

**Fig 3:**
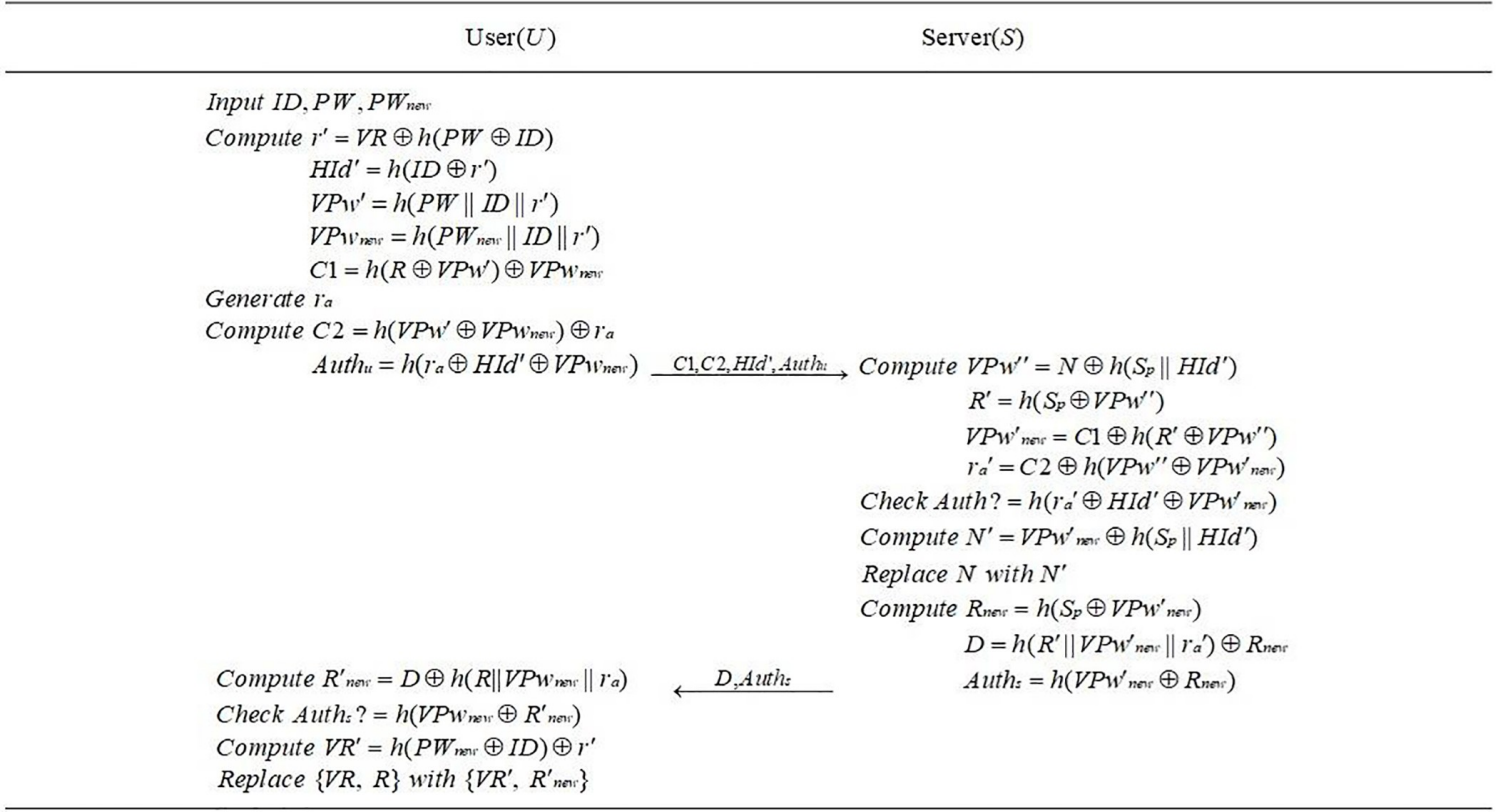
Password update phase.

In this section, in order to improve the security, we design an efficient provably secure mutual authentication scheme. Compared with the scheme of Qiu et al., our proposed scheme can resist various attacks and there is less pressure for users to remember. Our scheme consists of three phases: registration phase (see Fig 1), login and authentication phase (see Fig 2) and password update phase (see Fig 3). The proposed scheme is described as follows.

### 4.1| Registration phase

For a legal user *U*, if he or she wants to access the system, the necessary step is to register with the server *S* by submitting identity *ID* and password *PW*. User *U* and server *S* will perform the following steps.

1. *U* generates a long random number *r* and computes *VPw* = *h*(*PW*‖*ID*‖*r*) and *HId* = *h*(*ID*⊕*r*). Then *U* transmits {*VPw*,*HId*} to *S* by a secure channel.
2. After receiving the data, *S* computes *N* = *VPw*⊕*h*(*S*_*p*_‖*HId*) and *R* = *h*(*S*_*p*_⊕*VPw*). Then *S* stores {*HId*,*N*} into database and issues a smart card which contains *R* to *U* through a secure channel.
3. After receiving the smart card, *U* computes *VR* = *h*(*PW*⊕*ID*)⊕*r* and stores *VR* into the smart card.

### 4.2| Login and authentication phase

When a user *U* wants to acquire the service from server *S*, he or she should insert his or her smart card into card reader and enter his or her identity *ID* and password *PW*. *U* and *S* will perform the following steps.

1. The user *U* generates a random number *r*_*a*_, and computes *r*′ = *VR*⊕*h*(*PW*⊕*ID*), *HId*′ = *h*(*ID*⊕*r*′), *VPw*′ = *h*(*PW*‖*ID*‖*r*′), *C* = *h*(*R*⊕*VPw*′)⊕*r*_*a*_ and *Auth*_*u*_ = *h*(*r*_*a*_⊕*HId*′⊕*VPw*′). Then *U* transmits {*HId*′,*C*,*Auth*_*u*_} to the server *S*.
2. After receiving the message, *S* extracts *N* according to corresponding *HId*′ from the database and calculates *VPw*′′ = *N*⊕*h*(*S*_*p*_‖*HId*′), *R*′ = *h*(*S*_*p*_⊕*VPw*′′), *r*_*a*_′ = *h*(*R*′⊕*VPw*′′)⊕*C*. Then checks whether the value of *Auth*_*u*_ is equal to *h*(*r*_*a*_′⊕*HId*′⊕*VPw*′′). If holds, *S* picks a random number *r*_*b*_, computes *sk*_*s*_ = *h*(*r*_*a*_′‖*r*_*b*_‖*HId*′), *D* = *h*(*R*′‖*VPw*′′)⊕*r*_*b*_, *Auth*_*s*_ = *h*(*sk*_*s*_‖*D*) and transmits {*D*, *Auth*_*s*_} to *U*. Otherwise, *S* terminates the session.
3. When *U* receives the message, *U* computes *r*_*b*_′ = *h*(*R*‖*VPw*′)⊕*D*, *sk*_*u*_ = *h*(*r*_*a*_‖*r*_*b*_′‖*HId*′), checks whether *Auth*_*s*_ is equal to *h*(*sk*_*u*_‖*D*). If success, user *U* and server *S* share the same session key *sk*_*s*_ = *h*(*r*_*a*_′‖*r*_*b*_‖*HId*′) = *sk*_*u*_ = *h*(*r*_*a*_‖*r*_*b*_′‖*HId*′). If not, the smart card aborts the session.

### 4.3| Password update phase

If a user *U* needs to change password *PW* for a number of reasons, he or she only needs to input identity *ID*, password *PW* and new password *PW*_*new*_. User *U* and server *S* will perform the following steps.

1. The user *U* calculates *r*′ = *VR*⊕*h*(*PW*⊕*ID*), *HId*′ = *h*(*ID*⊕*r*′), *VPw*′ = *h*(*PW*‖*ID*‖*r*′), *VPw*_*new*_ = *h*(*PW*_*new*_‖*ID*‖*r*′) and *C*1 = *h*(*R*⊕*VPw*′)⊕*VPw*_*new*_. After generating a random number *r*_*a*_, *U* continue computing *C*2 = *h*(*VPw*′⊕*VPw*_*new*_)⊕*r*_*a*_, *Auth*_*u*_ = *h*(*r*_*a*_⊕*HId*′⊕*VP*_*Wnew*_). After that, *U* transmits {*C*1, *C*2, *HId*′, *Auth*_*u*_} to *S*.
2. Upon receiving the message, *S* computes *VPw*′′ = *N*⊕*h*(*S*_*p*_‖*HId*′), *R*′ = *h*(*S*_*p*_⊕*VPw*′′), *VPw*′_*new*_ = *C*1⊕*h*(*R*′⊕*VPw*′′) and *r*_*a*_′ = *C*2⊕*h*(*VPw*′′⊕*VPw*′_*new*_). Then *S* checks whether the value of *Auth*_*u*_ is equal to *h*(*r*_*a*_′⊕*HId*′⊕*VPw*′_*new*_). If not, *S* terminates this session, else continues to calculate *N*′ = *VPw*′_*new*_⊕*h*(*S*_*p*_‖*HId*′) to replace *N* with *N'* in the database. Finally, *S* computes *R*_*new*_ = *h*(*S*_*p*_⊕*VPw*′_*new*_), *D* = *h*(*R*′‖*VPw*′_*new*_‖*r*_*a*_′)⊕*R*_*new*_ and *Auth*_*s*_ = *h*(*VPw*′_*new*_⊕*R*_*new*_). After that, message {*D*, *Auth*_*u*_} will be transmitted to *U*.
3. After receiving the message {*D*, *Auth*_*s*_}, *U* gets *R'*_*new*_ by computing *R*′_*new*_ = *D*⊕*h*(*R*‖*VPw*_*new*_‖*r*_*a*_) and checks whether the value of *Auth*_*s*_ is equal to *h*(*VPw*_*new*_⊕*R*′_*new*_). If success, *S* computes *VR*′ = *h*(*PW*_*new*_⊕*ID*)⊕*r*′ and replaces {*VR*, *R*} with {*VR'*, *R'*_*new*_}, else terminates this session.

## 5 | Security analysis

In this part, we demonstrate our scheme is secure, practical and can provide kinds of security requirements. We assume that an adversary *A* might perform various attacks [18–21]. More detailed information is as follows.

### 5.1 |Insider attack

Assume that an adversary *A* can obtain *N* and *HId* of a legitimate user that stored in database. Because computational formulas are *HId* = *h*(*ID*⊕*r*), *N* = *VPw*⊕*h*(*S*_*p*_‖*HId*), *VR* = *h*(*PW*⊕*ID*)⊕*r*, *R* = *h*(*S*_*p*_⊕*VPw*) and *VPw* = *h*(*PW*‖*ID*‖*r*). Without the random number *r*, *A* cannot get the sensitive information *ID* or *PW* and cannot compute important secret data such as *R* and *VPw* form known data. Therefore, our scheme can resist insider attack.

### 5. 2|User anonymity

In the public channel, we do not send *Id* directly but transmit *HId* which is computed by means of the formula *HId* = *h*(*ID*⊕*r*). Even if *A* can access the value of *HId*, *A* still cannot get the sensitive information *Id*. Because the formula contains a random number *r* which *A* does not know. Therefore, our proposed scheme provides user anonymity.

### 5. 3|Replay attack

In our proposed scheme, the random numbers *r*_*a*_ and *r*_*b*_ change in every login. For an adversary *A*, he can intercept information {*HId'*, *C*, *Auth*_*u*_} and replay this message. Obviously, *A* can pass the verification of the server *S* and will receive the corresponding message {*D*, *Auths*} form sever *S*. Because *A* does not have a knowledge of the correct values *R*, *VPw* and *r*_*a*_, he cannot compute *r*_*b*_′ = *h*(*R*‖*VPw*′)⊕*D* and *sk*_*u*_ = *h*(*r*_*a*_‖*r*_*b*_′‖*HId*′). Therefore, our scheme is security even under replay attack.

### 5. 4| off-line password guessing attack

In our scheme, if an adversary *A* accesses exchanged information {*HId'*, *C*, *Auth*_*u*_, *D*, *Auth*_*s*_} which is transmitted in a public channel [22,23]. Because of the formula *HId*′ = *h*(*ID*⊕*r*′), *A* is unable to guess correct *ID* without *r*′. In addition, in the formulas *VPw*′ = *h*(*PW*‖*ID*‖*r*′), *C* = *h*(*R*⊕*VPw*′)⊕*r*_*a*_, *A* cannot guess *PW* correctly without the value *ID* and *r'*. Therefore, our scheme has an advantage of resisting off-line password guessing attack.

### 5. 5| Smart card lost attack

If an adversary *A* steals the smart card, he is able to get the *VR* and *R* which are stored in the smart card. Because the formulas are *VR* = *r*′⊕*h*(*PW*⊕*ID*) and *C* = *h*(*R*⊕*h*(*PW*‖*ID*‖*r*′))⊕*r*_*a*_, without the knowledge of *r’* and *r*_*a*_, *A* cannot guess the correct identity *ID* and password *PW*. If *A* wants to communicate with *S*, he needs to structure legitimate {*HId'*, *C*, *Auth*_*u*_}. Because of the formulas *HId*′ = *h*(*ID*⊕*r*′), *C* = *h*(*R*⊕*h*(*PW*‖*ID*‖*r*′))⊕*r*_*a*_, *Auth*_*u*_ = *h*(*r*_*a*_⊕*HId*′⊕*h*(*PW*‖*ID*‖*r*′)), without identity *ID* and password *PW*, *A* is unable to pass through the verification of server *S*. Therefore, our scheme can resist smart card lost attack.

### 5. 6| Impersonation attack

For an adversary *A*, in authentication phase, if he wants to masquerade as a legal user *U* and login in server *S*, he must forge a valid login message {*HId'*, *C*, *Auth*_*u*_}. It is impossible for *A* to forge valid login message without legitimate identity *ID*, password *PW*, *VR* and *R*. The same is true, if *A* wants to masquerade as the server *S*, he has to counterfeit message {*D*, *Auth*_*s*_}. Without valid information *N*, *A* is unable to obtain {*D*, *Auth*_*s*_} which can pass through verification of the user *U*. Furthermore, in password update phase, *A* is unable to forge valid *C1*, *C2* and *Auth*_*u*_ to pass the authentication of server *S*. In the same way, *A* is unable to forge valid *D* and *Auth*_*s*_ to pass the verification of user *U*. Therefore, our scheme can resist impersonation attack.

### 5. 7| Man-At-The-End attack

Man-At-The-End attack [24] contains widespread aspects and is difficult to model. The technical adversary is human that we call *A* here, he could authorize and limitless access to the target. All security protections stand up to *A* for a specific period of time. Because Man-At-The-End attack has concrete form in certain circumstance, one of the defense details is as follows:

*A* could personate a legitimate user to register and access the sensitive value of N* from the database end. His own identity ID*, password PW* are known. According to formulas *h*(*S*_*p*_‖*HId*) = *VPw*⊕*N*, *VPw* = *h*(*PW*‖*ID*‖*r*) and *HId* = *h*(*ID*⊕*r*). Without having knowledge about the value of random number *r*, he will take unpractical time cost to computer *S*_*p*_, by formulas *h*(*S*_*p*_‖*h*(ID*⊕*r*)) = *h*(PW*‖ID*‖*r*)⊕N*. Our scheme offer a defense and *A* is unable to obtain important information *S*_*p*_.

Although *A* could execute other forms and is hard to analyze, many protective devices have ability to against the attack, include software protection, hardware protection and digital asset protection, more details are reference no.24. Hence, our scheme can make a defense against Man-At-The-End attack.

### 5. 8| Perfect forward secrecy

When the system crashes in one session, an adversary can acquire information *ID*, *PW*, *S*_*p*_ and intercept message {*HId'*, *C*, *Auth*_*u*_} and {*D*, *Auth*_*s*_}. For an adversary *A*, he wants to get *sk*_*s*_ = *sk*_*u*_ = *h*(*r*_*a*_‖*r*_*b*_‖*HId*′). But without the knowledge of the correct values *VR* and *R*, he cannot compute *r*_*a*_′ = *h*(*R*′⊕*VPw*′′)⊕*C*, *r*_*b*_′ = *h*(*R*‖*VPw*′)⊕*D* correctly. Finally, *A* still have no ability to get the session key to eavesdrop session. Hence, our scheme provides perfect forward secrecy.

## 6 | Security proof

Security model: Burrows–Abadi–Needham logic (also known as the BAN logic) is a set of rules for defining and analyzing information exchange protocols [25]. Specifically, BAN logic helps its users determine whether exchanged information is trustworthy, secured against eavesdropping or both.

In this section, we will demonstrate that our scheme is secure and practical by the Burrows-Abadi-Needham (BAN) logic. We list some essential BAN-Logic symbols and formulas as follows, Table 2 introduces the notations of symbol and Table 3 introduces notations of formula. Supposing that P and Q are the symbols of participants, X and Y are statements as symbols, and K is the symbol for hash function key, next notations include more detailed explanation. We give reasoning process based on BAN-Logic in the following steps.

**Table 2:** Notations of symbol.

Symbol	Description
P|≡X	P believes X
P⇒X	P has jurisdiction over X
#(X)	X is fresh
P⊲X	P sees X
P|~X	P once said X
(X,Y)	X or Y belongs to (X, Y)
(X)_K_	X is hash with the key K
$P\overset{K}{↔}Q$	P communicates with Q using key K

**Table 3:** Notations of formula.

**Formula**	**Description**
$\frac{P|\equiv P\overset{K}{↔}Q,P⊲(X{)}_{K}}{P|\equiv Q|∼X}$	The message meaning rule
$\frac{P|\equiv \#(X)}{P|\equiv \#(X,Y)}$	The freshness rule
$\frac{P|\equiv \#(X),P|\equiv Q|∼X}{P|\equiv Q|\equiv X}$	The nonce-verification rule
$\frac{P|\equiv Q\Rightarrow X,P|\equiv Q|\equiv X}{P|\equiv X}$	The jurisdiction rule
$\frac{P|\equiv Q|\equiv (X,Y)}{P|\equiv Q|\equiv X}$	The believe rule

### Step 1 Our goals

In order to make our scheme practicable, we list some goals which need to be achieved.

Goal 1. $U|\equiv S|\equiv (U\overset{sk}{↔}S)$.

Goal 2. $U|\equiv (U\overset{sk}{↔}S)$.

Goal 3. $S|\equiv U|\equiv (U\overset{sk}{↔}S)$.

Goal 4. $S|\equiv (U\overset{sk}{↔}S)$.

### Step 2 Idealized form

We show the idealized message form between user *U* and server *S* as follows.

Msg 1. $U\to S:(r_{a},\;U\overset{r_{a}}{↔}S)VPw$.

Msg 2. $S\to U:(r_{a},\;r_{b},\;U\overset{r_{a}}{↔}S,\;U\overset{r_{b}}{↔}S,\;U\overset{sk}{↔}S)VPw$.

### Step 3 Initial state

We transform some premises about authentication and login phase in our scheme.

Aspt 1. *U*|≡#(*r*_*a*_).

Aspt 2. *S*|≡#(*r*_*a*_).

Aspt 3. $U|\equiv S\Rightarrow (U\overset{r_{b}}{↔}S)$.

Aspt 4. $S|\equiv U\Rightarrow (U\overset{r_{a}}{↔}S)$.

Aspt 5. $U|\equiv (U\overset{r_{a}}{↔}S)$.

Aspt 6. $S|\equiv (U\overset{r_{b}}{↔}S)$.

Aspt 7. $U|\equiv (U\overset{VPw}{↔}S)$.

Aspt 8. $S|\equiv (U\overset{VPw}{↔}S)$.

Aspt 9. $U|\equiv (U\overset{HId}{↔}S)$.

Aspt 10. $S|\equiv (U\overset{HId}{↔}S)$.

Aspt 11. $S|\equiv U|\equiv (U\overset{r_{b}}{↔}S)$.

Aspt 12. $S|\equiv U|\equiv (U\overset{HId}{↔}S)$.

### Step 4 Derivation process

We prove that user *U* and server *S* can set up the session key by combining with the above information.

(1) The proof of goal 1 and goal 2:

S 1.

On the basis of Msg 2, we get that $U⊲(r_{a},\;r_{b},\;U\overset{r_{a}}{↔}S,\;U\overset{r_{b}}{↔}S,\;U\overset{sk}{↔}S)VPw$, in the light of Aspt 7, we know that $U|\equiv (U\overset{VPw}{↔}S)$. Because of the message meaning rule, we get the following results:

$$U|\equiv S|∼(r_{a},\;r_{b},\;U\overset{r_{a}}{↔}S,\;U\overset{r_{b}}{↔}S,\;U\overset{sk}{↔}S).$$

S 2.

According to S 1 and Aspt 1, we know that $U|\equiv S∼(r_{a},\;r_{b},\;U\overset{r_{a}}{↔}S,\;U\overset{r_{b}}{↔}S,\;U\overset{sk}{↔}S)$ and *U*|≡#(*r*_*a*_). Owing to the nonce verification and the freshness rule, which is listed as follows.

$$U|\equiv S|\equiv (r_{a},\;r_{b},\;U\overset{r_{a}}{↔}S,\;U\overset{r_{b}}{↔}S,\;U\overset{sk}{↔}S).$$

S 3.

In line with S 2, we could achieve: $U|\equiv S|\equiv (U\overset{r_{b}}{↔}S)$ and

$$U|\equiv S|\equiv (U\overset{sk}{↔}S).$$

Goal 1 is realized apparently.

S 4.

According to $U|\equiv S|\equiv (U\overset{r_{b}}{↔}S)$ and Aspt 3 $U|\equiv S\Rightarrow (U\overset{r_{b}}{↔}S)$, By the jurisdiction rule, we confirmedly know that $U|\equiv (U\overset{r_{b}}{↔}S)$. According to $U|\equiv (U\overset{r_{b}}{↔}S)$, Aspt 5 $U|\equiv (U\overset{r_{a}}{↔}S)$, Aspt 9 $U|\equiv (U\overset{HId}{↔}S)$ and *sk* = *h*(*r*_*a*_‖*r*_*b*_‖*HId*), we make a representation is as follows:

$$U|\equiv (U\overset{sk}{↔}S).$$

So, we have achieved Goal 2.

(2) The proof of goal 3 and goal 4:

S 5.

From Msg 1, we know that $S⊲(r_{a},\;U\overset{r_{a}}{↔}S)VPw$, because of Aspt 8 $S|\equiv (U\overset{VPw}{↔}S)$ and the message meaning rule, we can deduce that:

$$S|\equiv U|∼(r_{a},\;U\overset{r_{a}}{↔}S).$$

S 6.

On the basis of S 5 and Aspt 2, we know $S|\equiv U|∼(r_{a},\;U\overset{r_{a}}{↔}S)$ and *S*|≡#(*r*_*a*_), owing to the nonce verification and the freshness rule, we obtain result what is as follows:

$$S|\equiv U|\equiv (r_{a},\;U\overset{r_{a}}{↔}S).$$

S 7.

According to S 6 and Aspt 4 $S|\equiv U\Rightarrow (U\overset{r_{a}}{↔}S)$, because of the jurisdiction rule, we could obtain $S|\equiv (U\overset{r_{a}}{↔}S)$. Form Aspt 6 $S|\equiv (U\overset{r_{b}}{↔}S)$, Aspt 10 $S|\equiv (U\overset{HId}{↔}S)$ and the formula *sk* = *h*(*r*_*a*_‖*r*_*b*_‖*HId*), we are able to get:

$$S|\equiv (U\overset{sk}{↔}S).$$

Goal 4 is accomplished.

S 8.

According to S 6 $S|\equiv U|\equiv (U\overset{r_{a}}{↔}S)$ and Aspt 11 $S|\equiv U|\equiv (U\overset{r_{b}}{↔}S)$, Aspt 12 $S|\equiv U|\equiv (U\overset{HId}{↔}S)$. Because of the formula *sk* = *h*(*r*_*a*_‖*r*_*b*_‖*HId*), we have following.

$$S|\equiv U|\equiv (U\overset{sk}{↔}S).$$

Distinctly, Goal 3 of our claim is achieved.

According to the proof above, our goals are achieved. We hold a sufficient reason to believe that both *U* and *S* believe that the session key *sk* which is shared between *U* and *S*.

## 7 | Performance comparison

**Fig 4:**
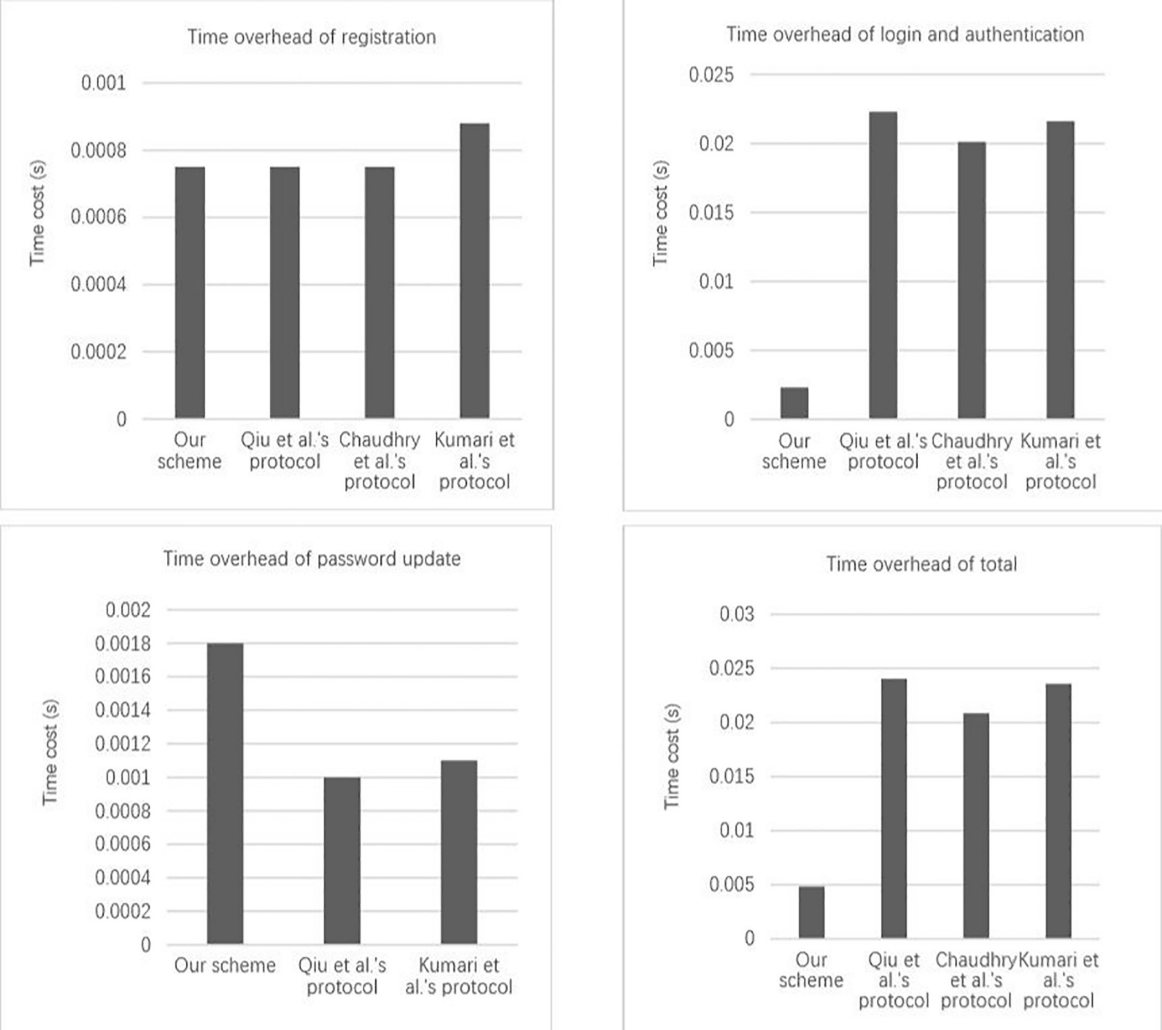
Time consumption comparison of other scholars' protocols and ours.

The experience of users play an important role in protocol. During this part, we will show a performance comparison between our scheme and the other schemes (see Fig 4). Before making a comparative analysis, we assume that one elliptic curve point multiplication operation is *T*_*pm*_, one hash function operation is *T*_*h*_. Other operations like generating a random number and exclusive-OR operation spend less time, which have little effect on performance comparison. So, we neglect the lightweight operations at this time. Before performance simulation test, we analysis performance of other protocols and ours in theory. So, we list Table 4 to descript theoretical time spend comparison.

**Table 4:** Comparison of our scheme and others.

Phase	Our scheme	Qiu et al.’s protocol [16]	Chaudhry et al.'s protocol [14]	Kumari et al.'s protocol [15]
Registration phase	*5T*_*h*_	*3T*_*h*_	*3T*_*h*_	*2T*_*h*_
Authentication phase	*15T*_*h*_	*12T*_*h*_ +6*T*_*pm*_	*7T*_*h*_ +6*T*_*pm*_	*10T*_*h*_ +*6T*_*pm*_
Password update phase	*19T*_*h*_	*10T*_*h*_		*8T*_*h*_
Total	*39T*_*h*_	*25T*_*h*_ +6*T*_*pm*_	*10T*_*h*_ +6*T*_*pm*_	*20T*_*h*_ +6*T*_*pm*_

From Table 4, at registration phase, execution time of our scheme mainly lies on executing 5*T*_*h*_. In other three protocols, execution time mainly lies on executing 3*T*_*h*_, 3*T*_*h*_ and 2*T*_*h*_. At authentication phase, execution time of our scheme mainly depends on executing 15*T*_*h*_. Meanwhile, execution time of other three protocols mainly depends on executing 12*T*_*h*_ +6*T*_*pm*_, 7*T*_*h*_ +6*T*_*pm*_, 10*T*_*h*_ +6*T*_*pm*_. At password update phase, execution time of our scheme mainly lies on executing 19*T*_*h*_. And the other two protocols’ execution time mainly depends on executing 10*T*_*h*_ and 8*T*_*h*_. The protocol of Chaudhry et al. lacks the phase of password update. In those four protocols, total execution time are 39 *T*_*h*_, 25 *T*_*h*_ +6 *T*_*pm*_, 10 *T*_*h*_ +6 *T*_*pm*_, 20 *T*_*h*_ +6 *T*_*pm*_ respectively.

In those four protocols, we find that our scheme performance mainly bases on hash function and the protocols of Qiu et al. [16], Chaudhry et al. [14] and Kumari et al. [15] are based on hash function and elliptic curve point multiplication. At registration phase and password update phase, we use a little more hash functions than those protocols. But the registration phase needs to be carried out only one time, so it has almost no effect on overall performance. And password update phase is not commonly used for a certain user, so it has little effect in practical applications. From authentication phase and total phases, though we use too many hash functions, other protocols all use six elliptic curve point multiplications. It is obvious that *T*_*pm*_ is many times as much as *T*_*h*_. Compared with other protocols, our scheme has a great advantage on computational costs in usual authentication phase and total phases.

We perform simulant performance comparison under the same computer simulation environment and write programs according to the schemes strictly. In the experiment, we run one hundred times to get the average data. According to the Fig 4, at registration phase, the time consumption in schemes of Qiu et al. [16], Chaudhry et al. [14] and we are all 0.00075s and Kumari et al.’s [15] scheme is 0.00088s. At password update phase, the time consumptions are 0.0018s, 0.001s, 0.0011s respectively. Although those scholars' protocols have a little bit better performance at registration phase and update phase, in practical application, those scholars' frequently-used authentication phases spend more time compared with our authentication phase. The costs of time are 0.0223s, 0.0201s, 0.0216s respectively, but ours is only 0.0023s. From comparative analysis, our total time is less than others. Obviously, our scheme is more efficient and more practice in application.

## 8 | Conclusion

In this paper, we review Qiu et al.’s protocol and find that it is vulnerable to some known attacks such as insider attack and denial service attack, then we review the scheme and carry on a strict security analysis about their scheme. Next, in order to solve these problems, we propose our more secure and more convenient scheme. Security analysis shows that our scheme can resist insider attack, off-line password guess attack and more, we give a sufficient reason. Then in security proof section, we adopt the BAN-logic to prove our scheme is secure and realizable. In the end, we make a performance comparison, the result shows that our scheme can be more suitable for users in SIP. Because under the same conditions, we can establish connections faster. In conclusion, compared to other protocols, our scheme is more security and practical.

## Supporting information

S1 Fig Transport details between user and server in registration phase. (TIF)

S2 Fig Transport details between user and server in login phase. (TIF)

S3 Fig Transport details between user and server in update phase. (TIF)

S4 Fig Performance Analysis Chart of the four schemes. (TIF)

S1 Code Zhang’s scheme. The VS code for analyzing the performance of the Zhang’s scheme.
(ZIP)

S2 Code Qiu’s scheme. The VS code for analyzing the performance of the Qiu’s scheme.
(ZIP)

S3 Code Kumari’s scheme. The VS code for analyzing the performance of the Kumari’s scheme.
(ZIP)

S4 Code Chaudhry’s scheme. The VS code for analyzing the performance of the Chaudhry’s scheme.
(ZIP)
