# Supplementary figures and images for: An improved and efficient mutual authentication scheme for session initiation protocol
Source: PLoS One. 2019 Mar 28;14(3):e0213688. doi: 10.1371/journal.pone.0213688 (PMC6438602; doi:10.1371/journal.pone.0213688)

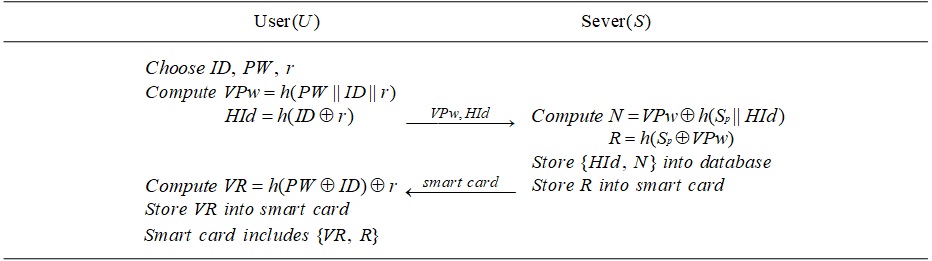

Supplement: S1 Fig — (TIF) [file pone.0213688.s001.tif]

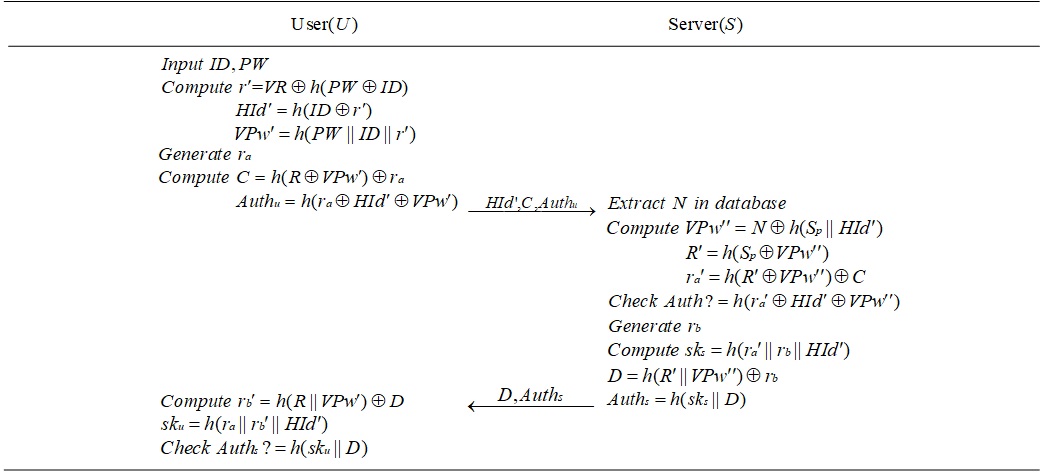

Supplement: S2 Fig — (TIF) [file pone.0213688.s002.tif]

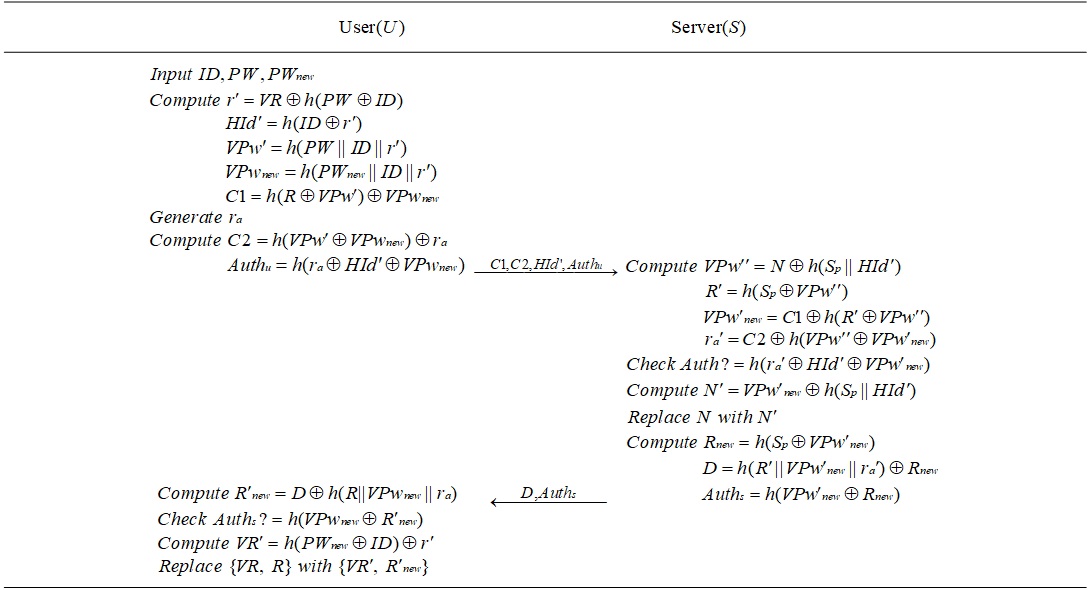

Supplement: S3 Fig — (TIF) [file pone.0213688.s003.tif]

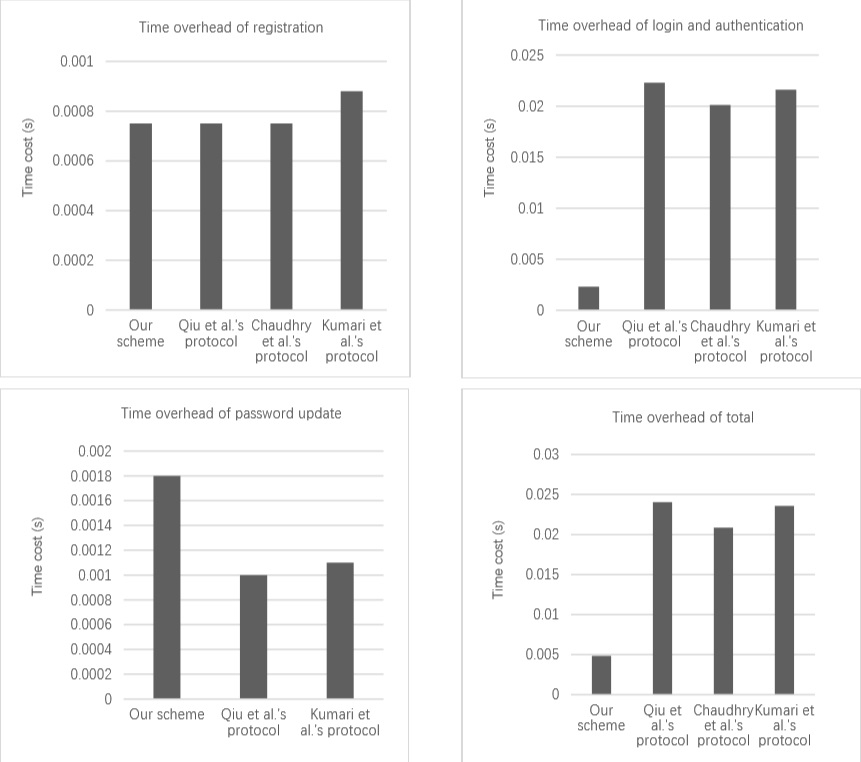

Supplement: S4 Fig — (TIF) [file pone.0213688.s004.tif]
